# Shear bond strength between alumina substrate and prosthodontic resin composites with various adhesive resin systems

**DOI:** 10.1186/s12903-015-0041-7

**Published:** 2015-05-02

**Authors:** Yousef A AlJehani, Jagan K Baskaradoss, Amrita Geevarghese, Marey A AlShehry, Pekka K Vallittu

**Affiliations:** Dental Health Department, College of Applied Medical Sciences, King Saud University, Riyadh, Saudi Arabia; Department of Dental Public Health, School of Dentistry, Case Western Reserve University, Cleveland, OH 44106 USA; College of Dentistry, King Saud Bin Abdulaziz University for Health Sciences, King Abdulaziz Medical City, Riyadh Saudi Arabia; Department of Biomaterials Science and Turku Clinical Biomaterials Centre, Professor and Chair of Biomaterials Science, Director of Turku Clinical Biomaterials Centre - TCBC, Institute of Dentistry, University of Turku and City of Turku, Welfare Division, Turku, Finland

**Keywords:** Adhesive resins, Prosthodontics resin composites, Shear bond strength

## Abstract

**Background:**

With the increase in demand for cosmetics and esthetics, resin composite restorations and all-ceramic restorations have become an important treatment alternative. Taking into consideration the large number of prosthodontic and adhesive resins currently available, the strength and durability of these materials needs to be evaluated. This laboratory study presents the shear bond strengths of a range of veneering resin composites bonded to all-ceramic core material using different adhesive resins.

**Methods:**

Alumina ceramic specimens (Techceram Ltd, Shipley, UK) were assigned to three groups. Three types of commercially available prosthodontic resin composites [BelleGlass®, (BG, Kerr, CA, USA), Sinfony® (SF, 3 M ESPE, Dental Products, Germany), and GC Gradia® (GCG, GC Corp, Tokyo, Japan)] were bonded to the alumina substrate using four different adhesive resins. Half the specimens per group (N = 40) were stored dry for 24 hours, the remaining were stored for 30 days in water. The bonding strength, so-called shear bond strengths between composite resin and alumina substrate were measured. Data were analysed statistically and variations in bond strength within each group were additionally evaluated by calculating the Weibull modulus.

**Results:**

Bond strengths were influenced by the brand of prosthodontic resin composites. Shear bond strengths of material combinations varied from 24.17 ± 3.72–10.15 ± 3.69 MPa and 21.20 ± 4.64–7.50 ± 4.22 at 24 h and 30 days, respectively. BG resin composite compared with the other resin composites provided the strongest bond with alumina substrate (*p* < 0.01). SF resin composite was found to have a lower bond strength than the other composites. The Weibull moduli were highest for BG, which was bonded by using Optibond Solo Plus adhesive resin at 24 h and 30 days. There was no effect of storage time and adhesive brand on bond strength.

**Conclusion:**

Within the limitations of this study, the shear bond strengths of composite resins to alumina substrate are related to the composite resins.

## Background

With the increase in demand for cosmetics and esthetics, resin composite restorations and all-ceramic restorations have become an important treatment alternative [1-3]. Recent developments in composites have expanded their clinical applications to prosthetic dentistry, and prosthodontic composites, namely veneering composites, are increasingly being used. These composites are used in the veneering of load-bearing substructures of fiber-reinforced composites and, in certain cases, for the adjustment and repair of ceramic restorations. In these applications, composites need to be joined to ceramic substructures [4,5].

Reinforced all-ceramic crowns consist of a high strength porcelain core material, laminated with dentin and incisal porcelain [6]. The all-ceramic restorations should have excellent physical properties, strength, marginal fit, and aesthetics necessary for anterior, as well as posterior, restorations [7]. Successful performance and reliability of veneered ceramic prostheses may be limited by mechanical integrity and adhesion of the veneering porcelain to the ceramic substrates. The mechanical properties of core materials and veneering porcelains should match to a certain extent to achieve a durable bond [8].

All-ceramic materials that can be utilized in load-bearing substructures include alumina (dialuminum trioxide) and yttria-stabilized zirconia (zirconium dioxide). Existing information is available about the adhesive properties of alumina and zirconia to composite resin luting cements using various surface conditioning methods [5,9-13]. It is known that alumina and zirconia ceramics with limited possibility to be roughened from their surface by hydrofluoric acid etching do not necessarily provide sufficient bond strength for composite resins. Adhesive failure between the framework and the luting cement has been reported in all-ceramic inlay-retained fixed partial dentures [14]. Studies have demonstrated that acidic monomer systems of composite resin luting cements and the use of a substrate-optimized surface primer may yield better adhesion to zirconia than neutral dimethacrylate monomer systems [8,15]. However, it has also been reported that the adhesion of composites based solely on chemical means is prone to the severe weakening hydrolytic effect of water under long-term water exposure [16].

The ceramic-composite bond is susceptible to chemical [17], thermal [18], and mechanical [19] influences under intraoral conditions. The simulation of such influences in the laboratory is compulsory to draw conclusions on the long-term durability of a specific bonding procedure and to identify superior materials and techniques. Without documented evidence of the strength of the bond between the core and veneering porcelain, the profession must rely on manufacturers’ claims to judge which material is best for patients.

The aim of this laboratory study was to evaluate the bonding strengths of a range of prosthodontic resin composites bonded to all-ceramic core material using different adhesive resins. The null hypothesis states that there is no difference in the shear strength when different prosthodontic resins are bonded to alumina using different adhesive resins.

## Materials and Methods

The lot numbers and manufacturers’ information for three types of prosthodontic resin composites and the four types of adhesive resins used in this study are listed in Table 1.

**Table 1:** **Materials used in this study**

**Description**	**Product**	**Lot No**	**Manufacture**
**Alumina substrate disc**	**Techceram**	Not Applicable	Techceram Ltd., Shipley, UK
**Veneering resin composites**	**Belle-Glass**	107373	Kerr Lab, Orange, CA 92867, USA
	**Sinfony®**	141365	3 M ESPE, Dental Products, Germany
	**GC-Gradia**	0209131	GC Corporation, Tokyo, Japan
**Adhesive resins**	**Scotchbond Multi-Purpose**	7543	3 M, Dental Products, MN 55144, USA
	**OptiBond Solo plus**	012851	Kerr Corporation, Orange, CA 92867, USA
	**Prime&Bond NT**	9810000585	Dentsply DeTrey, Konstanz, Germany
	**Stick Resin**	111686	Stick Tech Ltd., Turku, Finland

### Specimen preparation

All the alumina substrate discs (12 mm in diameter and 0.5 mm thickness) were supplied and fabricated by Techceram Limited (Shipley, UK). Each disc specimen was placed with the aesthetic surface down on a microscope glass slide in a Teflon ring mold (Φ = 12 mm). The molds were filled with a low-exothermic light-cured resin-composite. Care was taken during the embedding process to ensure that the test surface of the specimens was level with the edge of the mold. Brass rings (Φ = 14 mm) (University of Manchester Medical School Engineering Workshop, Manchester, England) were brushed with a separation agent (petroleum jelly) and then filled with dental stone. Each specimen was then mounted horizontally on the top of the filled brass rings. Microscope glass slides were used to bevel the discs embedded in the brass ring. During the mounting in stone, care was taken not to contaminate the prepared surfaces with the dental stone.

### Specimen organization into groups

Before the bonding procedures, the bonding surfaces were air-abraded with Rocatec® soft (3 M ESPE, Seefeld, Germany) for 60 s at 400 kPa. Pieces of Teflon (PTFE) [ICI, University of Manchester, UK] with circular holes of 6 mm in diameter and 3 mm thickness were prepared and attached to the specimen surface using double-sided adhesive tape [Sellotape, Switzerland] to determine the area of the bond.

The mounted alumina specimens were randomly assigned to three groups. The specimens were prepared for bonding with three different veneering composite resin using four different bonding resins, to be tested after 24 hours water storage and 30 days water storage and the temperature was maintained at 37°C.

### Bonding procedures

The procedures for materials handling and application were performed at room temperature and 50% humidity. All the adhesives and materials were applied to the alumina surface. The information on the organization of the specimens for different prosthodontic resin composites are shown in Table 2. The groups were as follows:

**Table 2:** **Organization of the specimens for different resin composites**

	**Storage time (24 hours)**		**Storage times (30 days)**	
**Group 1**	**Belle-Glass resin composite**			
	Scotchbond Multi-Purpose	10	Scotchbond Multi-Purpose	10
	OptiBond Solo plus	10	OptiBond Solo plus	10
	Prime & Bond NT	10	Prime & Bond NT	10
	Stick Resin	10	Stick Resin	10
		**N = 40**		**N = 40**
**Group 2**	**Sinfony Resin Composite**			
	Scotchbond Multi-Purpose	10	Scotchbond Multi-Purpose	10
	OptiBond Solo plus	10	OptiBond Solo plus	10
	Prime & Bond NT	10	Prime & Bond NT	10
	Stick Resin	10	Stick Resin	10
		**N = 40**		**N = 40**
**Group 3**	**GC Gradia Resin Composite**			
	Scotchbond Multi-Purpose	10	Scotchbond Multi-Purpose	10
	OptiBond Solo plus	10	OptiBond Solo plus	10
	Prime & Bond NT	10	Prime & Bond NT	10
	Stick Resin	10	Stick Resin	10
		**N = 40**		**N = 40**

####  Group 1

The adhesive resin was applied to the bonding surface of alumina, followed by the application of BelleGlass® (BG) via the hole in the Teflon mold. The required amount of material was further compressed and smoothed with a plastic instrument, and then, both the adhesive and BG were light cured at the same time from the top of the mold with a BG light curing unit for 40 s, followed by heat and pressure curing for 20 min. A temperature of 120°C and a pressure of 414 kPa N_2_ were applied using a BG HP heat-pressure curing oven.

####  Group 2

The adhesive resin was applied to the bonding surface of alumina, followed by application of the prosthodontic composite Sinfony® (SF) via the hole in the Teflon mold. Then, both the adhesive and SF resin composite were light cured at the same time from the top of the mold with a BG light curing unit for 40 s, followed by photopolymerization (Visio Beta Vario light curing unit) up to 40°C, vacuum for 15 minutes with a maximum temperature of 70°C.

####  Group 3

The adhesive resin was applied to the bonding surface of alumina, followed by application of the composite resin GC Gradia® (GCG) via the hole in the Teflon mold. Both the adhesive and GCG resin composite were light cured at the same time from the top of the mold with a BG light curing unit for 40 s, followed by photopolymerization using a GC Light-cure Labolight LVIII unit for 3 minutes and finally heat-cured at 100–110°C for 15 min in a Petit Oven PO-I (GC Corp).

### Shear bond strength testing

The alumina and prosthodontic composite's bonding strength, so-called shear bond strength test was performed using a Howden Universal Testing Machine (Leamington Spa, England) running at a cross-head speed of 0.5 mm/minute. A circular knife-edged blade was used to deliver a shearing force parallel to the bonded surfaces. To measure the bond strength, which was the measure of durability of adhesion between the materials regardless of the location of the failure, the bonded specimen was mounted in a jig attached to a 500-N load cell in the testing machine. The calculated bond strength was determined by dividing the force at which bond failure occurred by the bonding area [20]. The peak force in N was recorded when the bond failure occurred and noted immediately after testing.

### Microscopic evaluation

Following the shear bond strength testing, each specimen was inspected visually under a Wild M3Z light microscope (Wild Heerbrugg Ltd., Heerbrugg, Switzerland) to examine the failure area. All the specimens were then examined at 20X magnification to determine the location and type of failure that had occurred during the debonding procedure. The type of failure was categorized as follows:

A: Adhesive failure (failure that occurred between the veneering resin and the surface of the alumina substrate).
B: Cohesive failure (failure that occurred within the alumina substrate or within the veneering composite).
C: Mixed failure (adhesive and cohesive fracture of the material, with part of the failure remaining on the alumina substrate).

### Statistical analysis

The data were analyzed by two-way ANOVA using the SPSS (Statistical Package for the Social Sciences). The mean and standard deviation for the bond strength were calculated for each group. Scheffe’s multiple comparison test was used to detect differences in the shear bond strength among groups of different materials and within groups.

The strength variations within each group were evaluated by calculating the Weibull modulus (*m*). An Excel® spreadsheet was used to rank the shear strength data in ascending order and appoint a rank over the range 1 to N (N is the number of specimens); a straight line was then fitted through the points using the median rank regression method. The following equation was used to calculate the Weibull modulus:

$$ {P}_f=1\hbox{--} exp\left[\hbox{--} {\left(\sigma /{\sigma}_0\right)}^m\right], $$

where *P*_*f*_ is the failure probability, σ is the strength at a given *P*_*f*_, σ_*0*_ is the characteristic strength and *m* is the Weibull modulus. However because *P*_*f*_ can be identified by the following relation

$$ {P}_f=j/\left(N+1\right), $$

where j is the rank in strength and N is the number of specimens, equation 1 can be rewritten as

$$ 1/\left(1\hbox{--} {P}_f\right)=1/ exp\left[\hbox{--} {\left(\sigma /{\sigma}_0\right)}^m\right)\Big]. $$

Accordingly, ln [1/(1─ *P*_*f*_)] vs. ln (strength) will yield a slope equal to the Weibull modulus (m) [21,22]. Weibull analysis was also used to predict the failure probability at any level of stress from which the reliability or predictability of the shear bond strength could be quantified.

## Results

The bond strengths were affected by the brand of prosthodontic resin composites. The shear bond strengths of material combinations after 24 hours and 30 days of water storage varied from 24.17 ± 3.72 to 10.15 ± 3.69 MPa and from 21.20 ± 4.64 to 7.50 ± 4.22, respectively (Table 3). BG bonded to alumina using Optibond Solo Plus adhesive resin at 24 h and 30 days exhibited the highest shear bond strength followed by the GCG composite resin bonded to Techceram using Optibond Solo Plus adhesive resin. The SF veneering material applied to the alumina substrate exhibited the weakest bond strength (10.15 ± 3.69 MPa after 24 h and 7.50 ± 4.22 MPa after 30 days). The differences between the brands of prosthodontic composites were statistically significant (P < 0.01). There was no significant difference in the shear bond strength values between the two storage periods, i.e., 24 hours in water or 30 days in water (P = 0.62). Additionally, there was no significant difference in the shear bond strengths between the different types of adhesive resins (P = 0.09). The statistical analysis results of the shear bond strengths are shown in Table 4.

**Table 3:** **Shear bond strength values of different composite resins bonded to alumina substrate using different bonding resins**

	**Storing criteria**			
	**24 h of water storage at 37°C**		**30 days of water storage at 37°C**	
**Veneering resin and adhesive Resin**	**Bond strength (MPa) ± SD**	**Mode of failure (Adhesive = A Cohesive = C)**	**Bond strength (MPa) ± SD**	**Mode of failure (Adhesive = A Cohesive = C)**
**BG & Scotchbond Multipurpose bonding resin**	17.1 ± 3.6	A = 3	13.9 ± 4.9	A = 5
		C = 7		C = 5
**BG & Prime & Bond NT bonding resin**	14.6 ± 2.7	A = 5	11.3 ± 4.9	A = 4
		C = 5		C = 6
**BG & OptiBond Solo plus bonding resin**	24.2 ± 3.7	A = 2	21.2 ± 4.6	A = 4
		C = 8		C = 6
**BG & Stick Resin Adhesive bonding resin**	13.2 ± 3.9	A = 4	10.7 ± 4.7	A = 5
		C = 6		C = 5
**SF & Scotchbond Multipurpose bonding resin**	14.6 ± 2.8	A = 9	11.7 ± 4.3	A = 10
		C = 1		C = 0
**SF & Prime & Bond NT bonding resin**	11.8 ± 3.0	A = 10	8.5 ± 3.9	A = 10
		C = 0		C = 0
**SF & OptiBond Solo plus bonding resin**	19.1 ± 3.2	A = 9	14.6 ± 5.0	A = 10
		C = 1		C = 0
**SF & Stick Resin Adhesive bonding resin**	10.2 ± 3.7	A = 10	7.5 ± 4.2	A = 10
		C = 0		C = 0
**GCG & Scotchbond Multipurpose bonding resin**	16.0 ± 4.6	A = 9	13.5 ± 4.4	A = 10
		C = 1		C = 0
**GCG & Scotchbond Prime & Bond NT bonding resin**	13.4 ± 3.8	A = 10	10.4 ± 5.0	A = 10
		C = 0		C = 0
**GCG & OptiBond Solo plus bonding resin**	23.1 ± 4.2	A = 8	19.0 ± 7.4	A = 7
		C = 2		C = 3
**GCG & Stick Resin Adhesive bonding resin**	12.1 ± 3.6	A = 10	9.4 ± 3.2	A = 10
		C = 0		C = 0

BG - Belle-Glass; SF - Sinfony; GCG - GC Gradia.

**Table 4:** **Statistical analysis of shear bond strengths of resin composites bonded to alumina substrate at different periods of water storage (24 h, 30 days) followed by Scheffe’s Multiple Comparison test**

**Materials**		**Mean difference**	**P value**	**95% confidence interval**	
				**Lower bound**	**Upper bound**
Belle-Glass	Sinfony	3.53	<0.01	1.80	5.26
	GC Gradia	1.15	0.26	-0.58	2.88
Sinfony	Belle-Glass	−3.53	<0.01	−5.26	−1.80
	GC Gradia	−2.38	<0.01	−4.11	−0.64
GC Gradia	Belle-Glass	−1.15	0.26	−2.88	0.58
	Sinfony	2.38	<0.01	0.64	4.11

**Figure 1:**
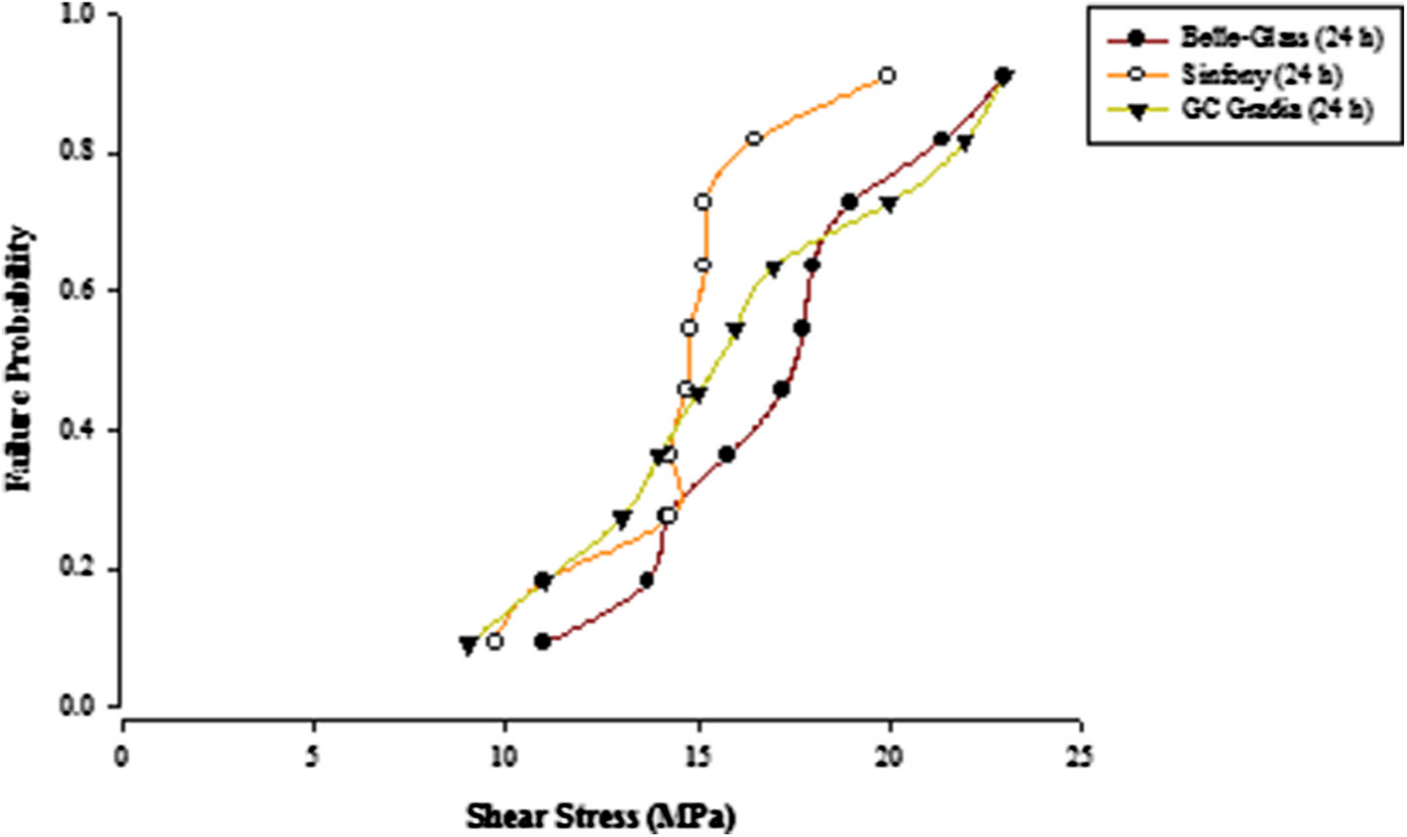
Probability of failure versus shear stress for different composites bonded to alumina substrate using Scotchbond Multipurpose (Storage time 24 h).

**Figure 2:**
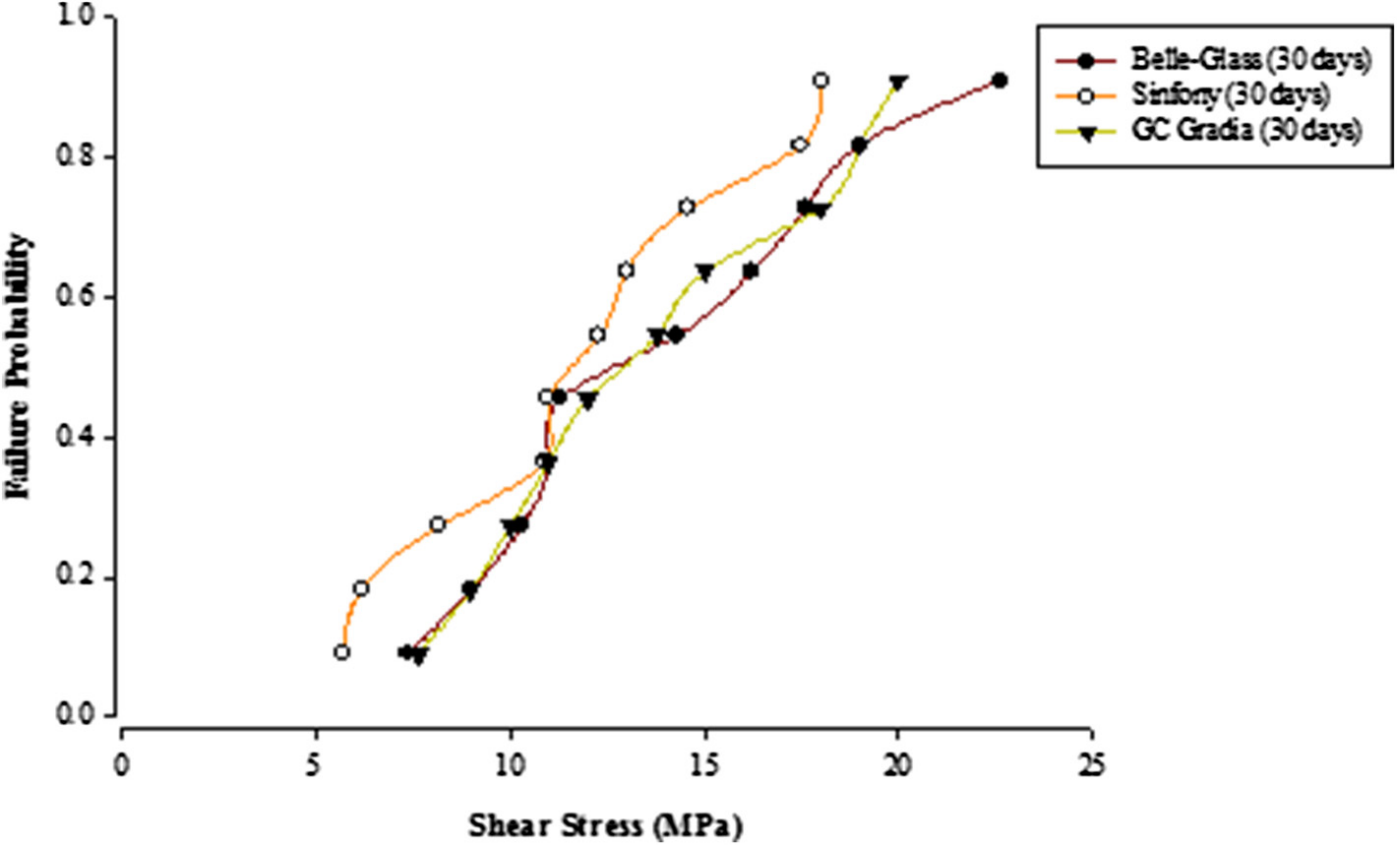
Probability of failure versus shear stress for different composites bonded to alumina substrate using Scotchbond Multipurpose (Storage time 30 days).

**Figure 3:**
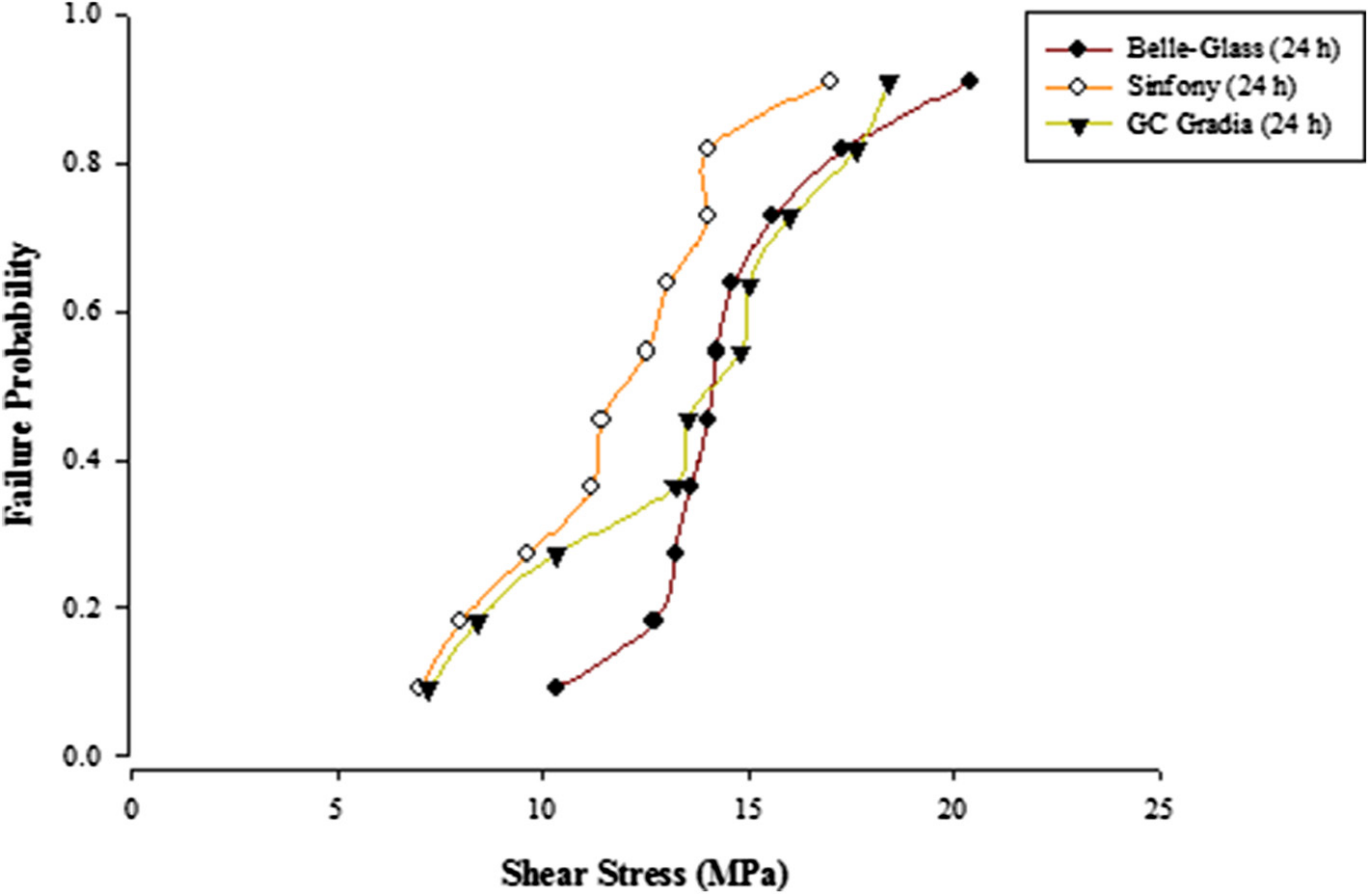
Probability of failure versus shear stress for different composites bonded to alumina substrate using Prime & Bond NT (Storage time 24 h).

**Figure 4:**
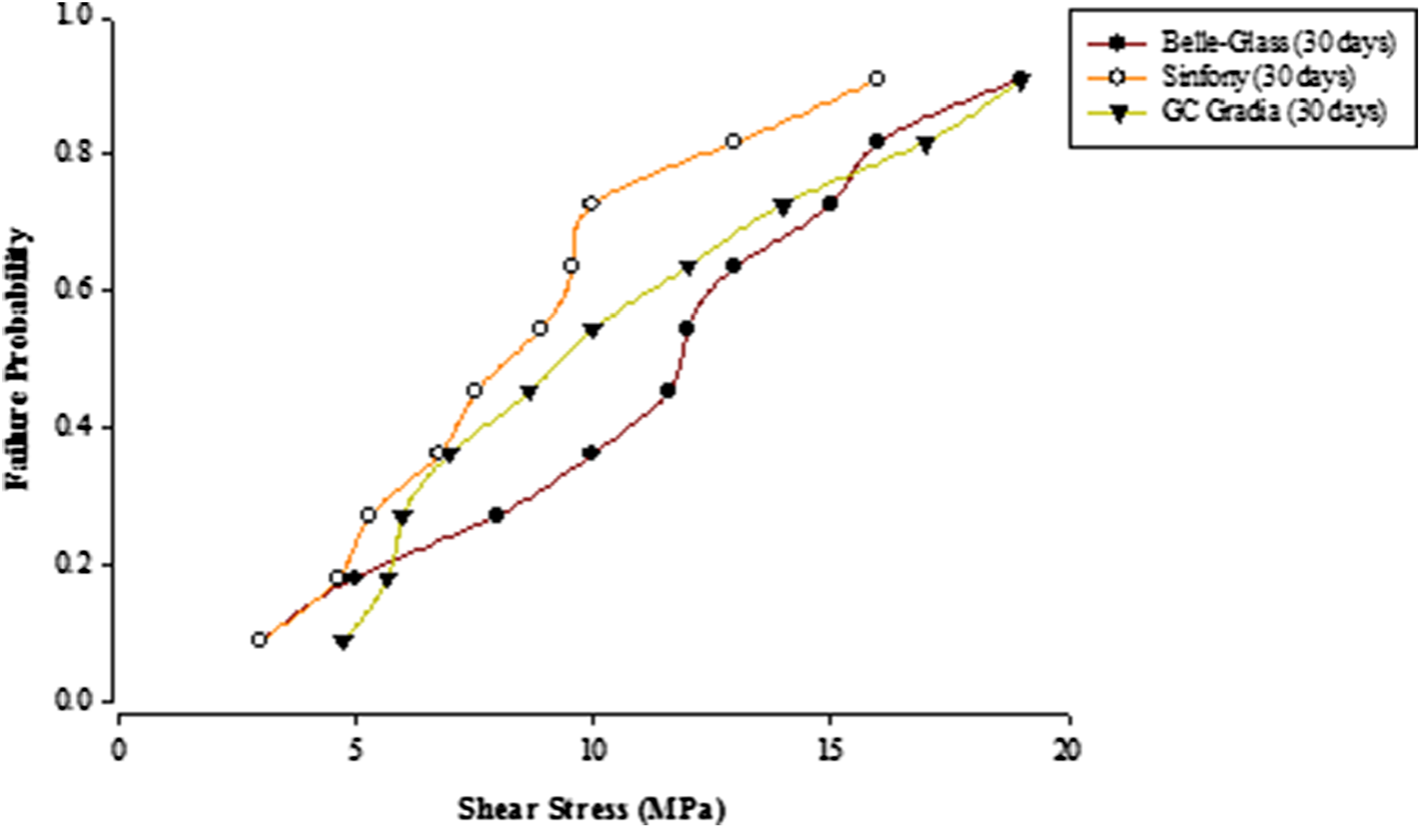
Probability of failure versus shear stress for different composites bonded to alumina substrate using Prime & Bond NT (Storage time 30 days).

**Figure 5:**
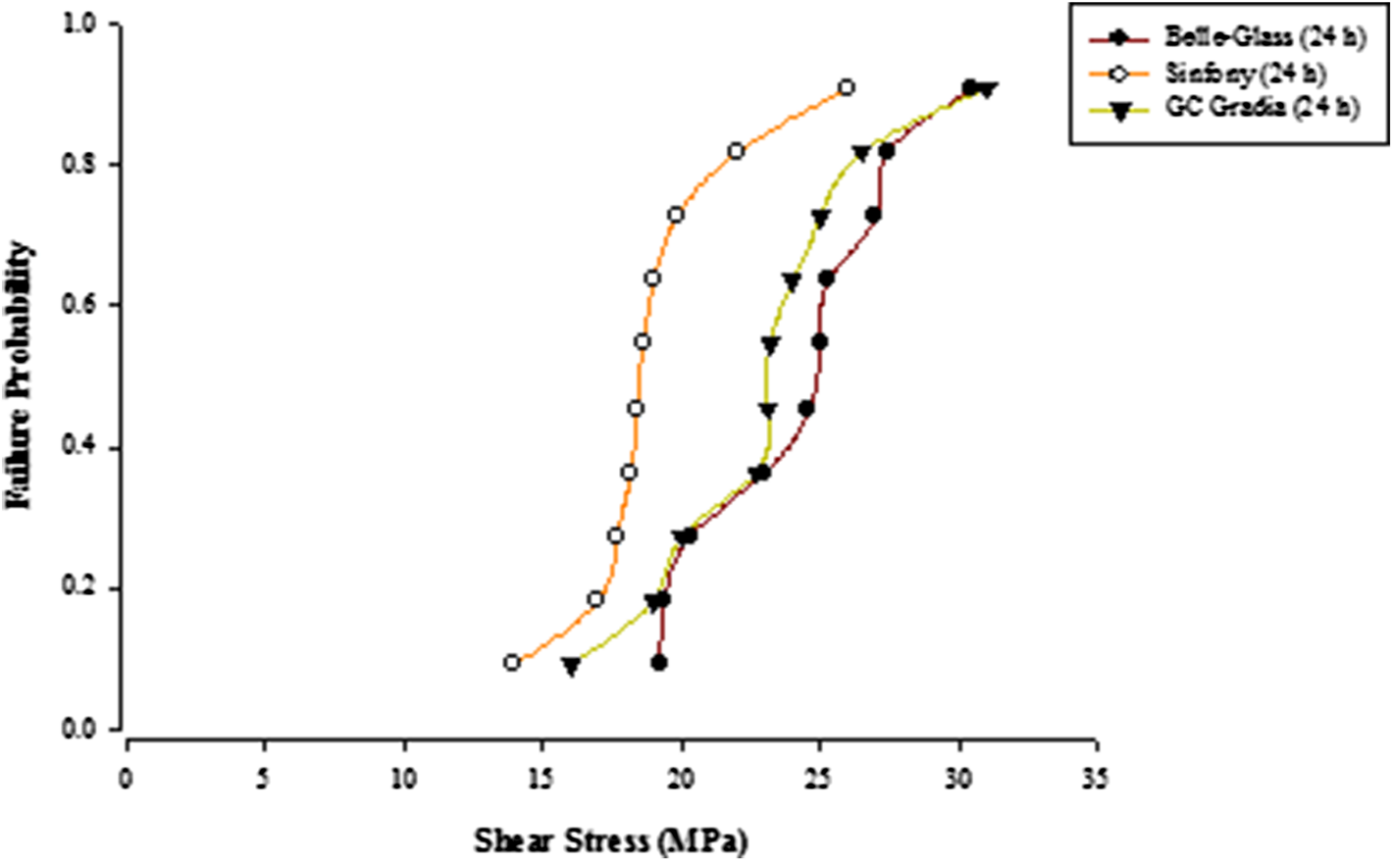
Probability of failure versus shear stress for different composites bonded to alumina substrate using OptiBond Solo Plus (Storage time 24 h).

**Figure 6:**
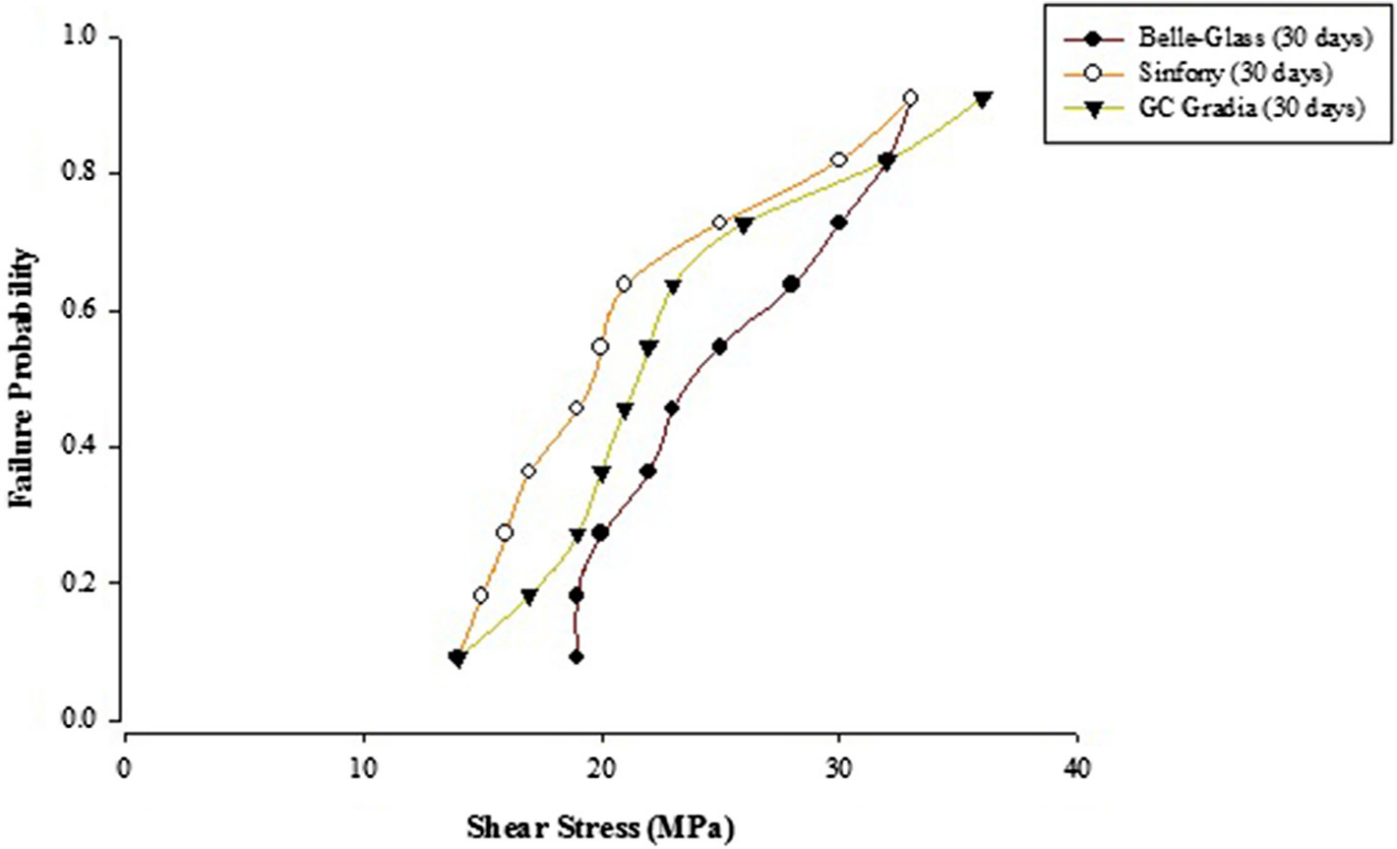
Probability of failure versus shear stress for different composites bonded to alumina substrate using OptiBond Solo Plus (Storage time 30 days).

**Figure 7:**
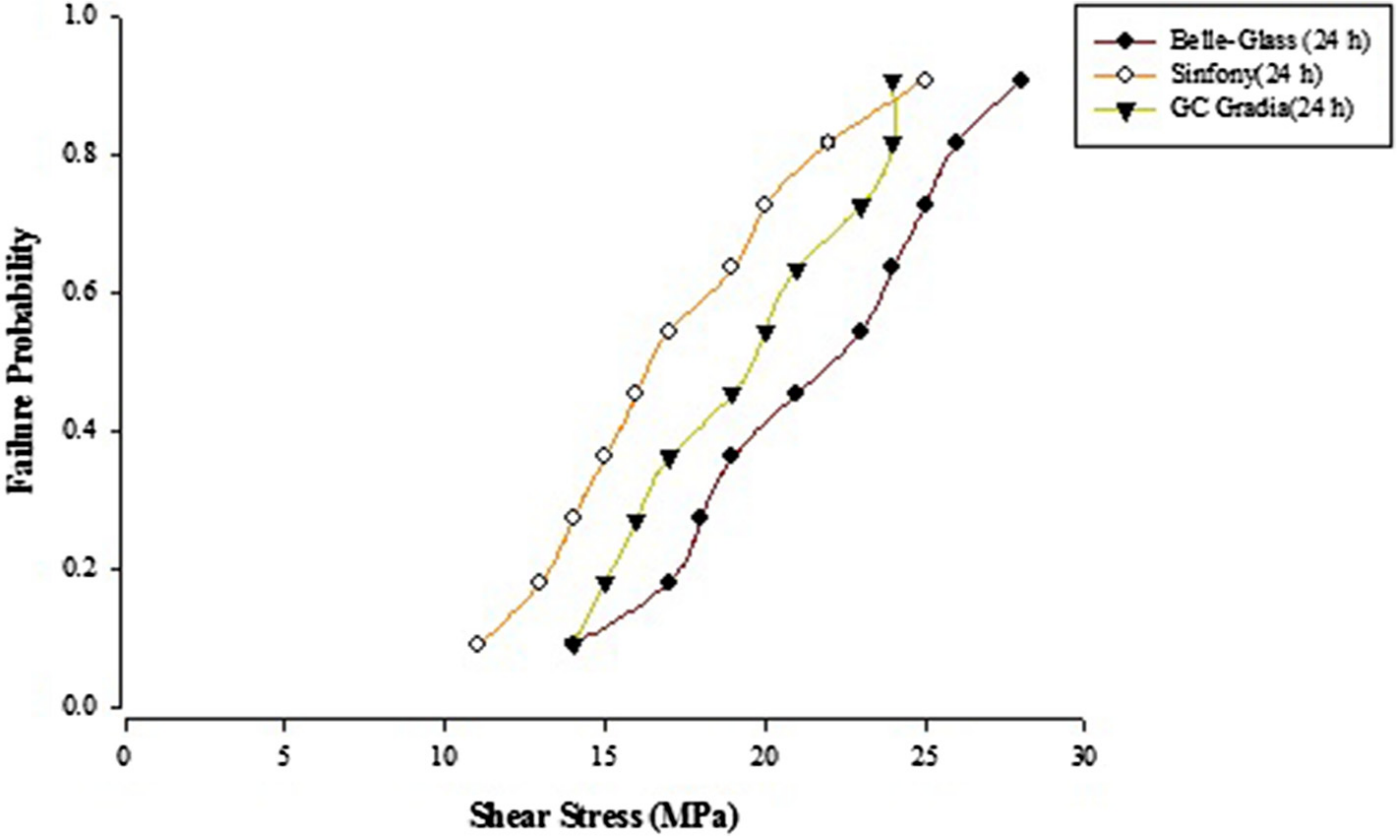
Probability of failure versus shear stress for different composites bonded to alumina substrate using Stick resin (Storage time 24 h).

**Figure 8:**
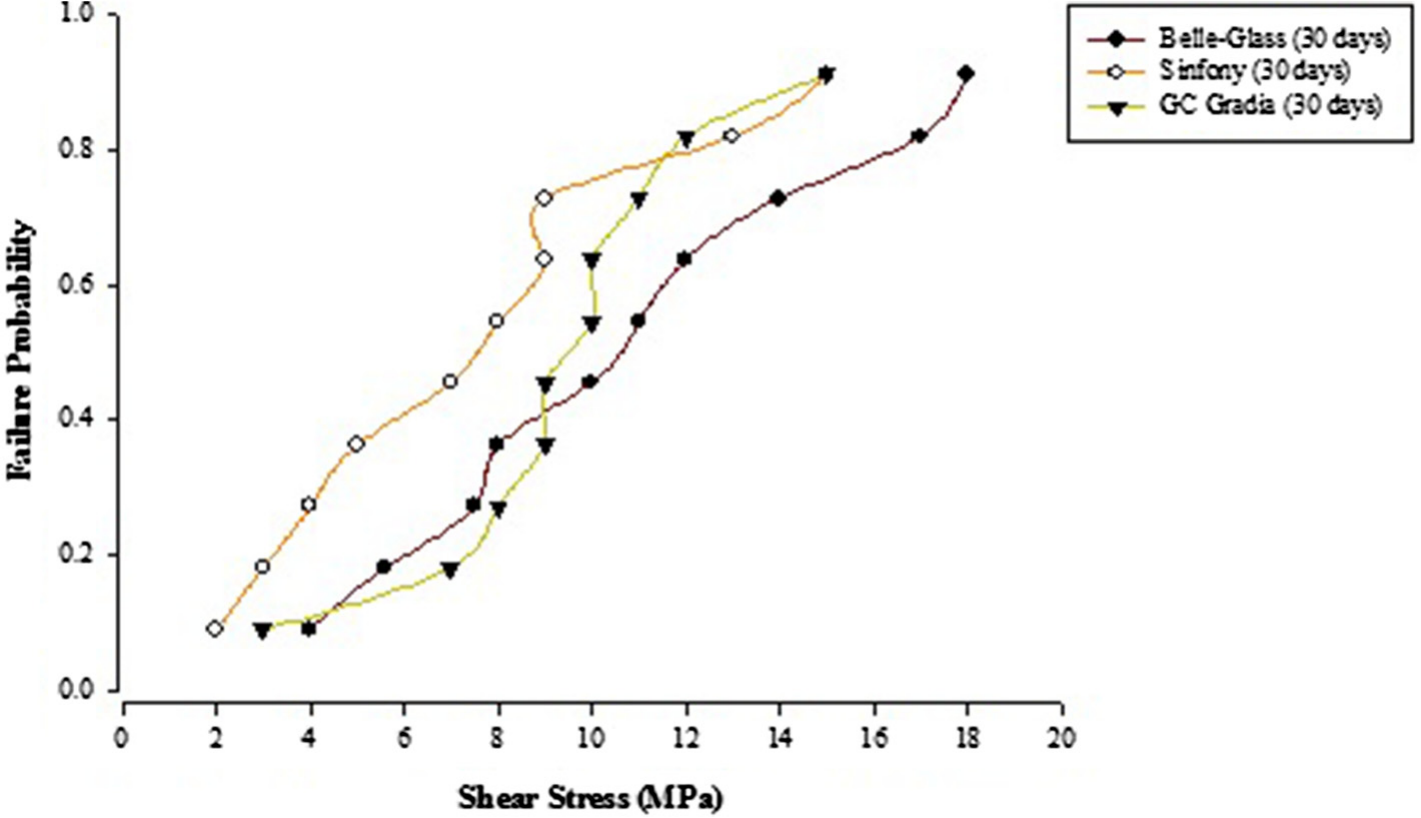
Probability of failure versus shear stress for different composites bonded to alumina substrate using Stick resin (Storage time 30 days).

When the failure sites were examined, two types of failures were observed. In general, the adhesive type of failure was more dominant than the cohesive type for all the prosthodontic composites resins, surface conditioning methods and water storage times. The bond strength data for the prosthodontic resin composites bonded to alumina substrates prepared with the different adhesive resins were further analyzed using the Weibull distribution function. Weibull analysis was used to predict the failure probability at any level of stress and generate a value of the Weibull modulus from which the reliability or predictability of the bond could be quantified. The probability of failure versus shear stress for different prosthodontic composites bonded to alumina substrates using different adhesive resins is plotted in Figures 1, 2, 3, 4, 5, 6, 7 and 8.

Weibull analysis for different prosthodontic composites bonded to alumina substrates using different adhesive resins is shown in Table 5. The Weibull moduli were highest for BG bonded to alumina using Optibond Solo Plus adhesive resin at 24 h and 30 days. ScotchBond Multipurpose adhesive resin with GCG resin and Stick Tech adhesive resin with SF exhibited the lowest Weibull moduli at 24 h and 30 days, respectively.

**Table 5:** **Weibull modulus analysis of shear bond strengths for resins composites bonded to alumina substrate with various bonding resins**

**Bonding resins**	**Belle-Glass (95% CI)**		**Sinfony (95% CI)**		**GC Gradia (95% CI)**	
	**24 h**	**30 days**	**24 h**		**24 h**	**30 days**
**ScotchBond Multipurpose**	4.5 (0.2)	2.8 (0.1)	4.7 (0.6)	2.5 (0.6)	1.2 (0.2)	3.0 (0.6)
**Prime and Bond**	5.2 (0.3)	1.7 (0.3)	3.7 (0.2)	2.0 (0.6)	3.1 (0.2)	2.0 (0.1)
**OptiBond Solo Plus**	6.2 (0.3)	4.5 (0.3)	5.8 (0.3)	2.8 (0.5)	5.3 (0.3)	2.4 (0.5)
**Stick Tech Adhesive**	3.3 (0.3)	2.1 (0.5)	2.8 (0.4)	1.0 (0.6)	3.3 (0.2)	2.2 (0.4)

## Discussion

The variation in the bond strength between the alumina substrate and three types of prosthodontic composite resins using different adhesive resins was investigated. The results indicated that the shear bond strength of a prosthodontic composite resin to alumina was affected by the type of composite used.

Bond strength measurements are among the methods used to evaluate the effectiveness of adhesive systems, hence predicting their performance in the oral environment. The efficacy of bonding agents is mainly evaluated by shear and/or tensile bond strength measurements [23]. Shear bond testing is one of the most popular tests used to evaluate bond strength, although it has been used and criticized especially in dentine bonding applications [24-27]. The shear bond strength test is defined as a test in which two materials are connected via an adhesive agent and loaded in shear until separation occurs [28]. This test is relatively simple and easy to perform, producing rapid results. In shearing, the bond is broken by a force parallel to the tooth surface. However, in tension, the bond is broken by a force perpendicular to the tooth surface. The shear strength is then calculated by dividing the maximum applied force by the bonded cross-sectional area [20,29]. This measurement provides information about the adhesive behavior of various types of materials and can be considered as a screening test [30].

An evaluation of the mode of failure of the specimens is important in demonstrating the quality of the bond to treated ceramic surfaces and prosthodontic composites resins. In this experiment, it was noted that the tested specimens exhibited more adhesive type failures than cohesive failures. However, many investigations have reported that the mode of failure occurring after shear bond testing is often cohesive within the substrate or bonded composite rather than adhesive at the interface [31,32]. Testing the bond strength by tensile loading produces more adhesive failures, which may favor the evaluation of the true bond strength [33]. However, the results are greatly affected by the specimen geometry and the occurrence of non-uniform stress distributions during load application [34].

The relative values of bond strength may be accountable for the modes of failure at the bonded interface [35-37]. Cohesive failure is usually recognized to increase the bond strength values because of the fracture propagation through the bulk material of a bonded material. Cohesive failure was evidenced by the multiple fractured tags of BG resin composite retained in the undercuts on the alumina surface; however, this type of failure was not distinct in the alumina surface bonded to the GCG and SF prosthodontic composite resins. In fact, the fracture surface feature of alumina was smooth to observe the fractured composite remnants.

Adhesive failure does not occur in the presence of a good bond between a compatible ceramic core and veneer material. Microscopic examination of the surface of the alumina substrate bonded to the BG resin composite revealed that the failure occurred at the bulk of the material, with residue of the veneering composite remaining on the core. According to Oden et al. [38], the strength of the veneering material in combination with the alumina coping was demonstrated to be excellent. These authors reported that veneering materials are chemically bonded to the densely sintered aluminum oxide by ionic and covalent bonds.

In general, the tested prosthodontic composite resins with alumina substrates revealed considerable variations in the shear bond strength between the different prosthodontic composites. This finding could be attributed to the differences in the individual properties of the materials. The incidence of cohesive failure was greater in the alumina surfaces bonded the BG composite resin compared than the others. However, the SF and GCG resin composites are only light-cured composites and exhibited an adhesive mode of failure; therefore, heat treatment of BG may increase the bond strengths to the Techceram substrate. It is likely that the increased polymerization temperature of the BG composite enhanced the polymerization of the adhesive resin to the surface of alumina, which results in higher bond strength values. Cohesive type failure occurred in the specimens with high shear bond strengths. Fortin et al. [39] also reported that cohesive failures tended to occur in adhesive materials exhibiting high shear bond strengths. Barkmeier et al. [40] reported that when a cohesive fracture occurs, a substantial amount or area of the substrate material is sheared off, thus requiring a much higher shearing load to fracture.

All the data obtained for the shear bond strength of different indirect prosthodontic composites was subjected to Weibull analysis. The use of Weibull distributions has proved to be a good method for the evaluation of the fracture behavior of materials [41]. This distribution is used where bond strength measurements and predictions are important. In addition, this distribution predicts the probability of bond failure under a specific stress value. However, it have to be highlighted that the sample size of this study limits the reliability of the Weibull analysis and therefore results can only be considered as indicative only. BG resin composite compared with the other prosthodontic resin composites provided a more durable bond to alumina. This composite was also observed to have a high likelihood of resisting fracture at low and high stress levels. The SF veneering material applied to alumina resulted in a weaker bond compared with the other veneering composites. The SF composite was polymerized at the lowest polymerization temperature of the tested prosthodontic composites, which may explain the lower bond strength values.

The results of the present work indicate no significant difference in the shear bond strengths between the different types of adhesive resins, which can be understood by their chemical compositions. The adhesive resins used in this study predominantly did not contain acidic monomers, except Prime&Bond, which may have demonstrated some variation in relation to the adhesive resins, as demonstrated by Cooley et al., who observed that the bond strength of composite resin to dental ceramic is affected by the bonding agent and the type of composite resin [42]. Gregory and Moss [43] reported that the hybrid composite resins generally provide higher bond strengths than microfilled composite resins.

An interesting finding was that there was no statistically significant difference in the shear bond strength values between the two storage periods, namely the 24 hours and 30 days water storage periods, although there was a trend for lower values for the specimens stored for 30 days. More aggressive tests using longer periods of water storage or thermal cycling could have reduced the bond strength values even more. The temperature was maintained at 37°C to ensure that the environmental conditions did not affect the physical properties of the resin [44,45]. Further studies are needed to study the effect of the temperature of polymerization of the composite resins, adhesives and primers to the surface of ceramics to enhance the short-term and long-term durability of the adhesive joint between the composites and ceramics.

## Conclusions

Within the limitations of this study, the shear bond strengths of composite resins to alumina substrate are related to the composite resins. However, there was no effect of storage time and adhesive brand on the bond strength.
